# Effect of Eruca sativa on Spermatogenesis in Rats Exposed to Cigarette Smoke

**DOI:** 10.7759/cureus.67662

**Published:** 2024-08-24

**Authors:** Chro G Raouf, Ahmed O Noori, Khoshawist Y Salih, Tre H Mohammad, Awyar R Mohammed, Jihad M Hadi

**Affiliations:** 1 Medical Laboratory of Science, College of Health Sciences, University of Human Development, Kurdistan Regional Government, Sulaimani, IRQ

**Keywords:** eruca sativa, cigarette smoking, spermatogenesis, sex hormones, lipid profile

## Abstract

Smoking is among the significant adverse factors to reproductive health and accounts for damage to spermatogenesis and maturation of spermatozoa. The proposed research contributes to understanding the potential of *Eruca sativa* to prevent the cytotoxic effect of tobacco smoke on different aspects of male reproductive health, including sperm: sperm morphology, sperm count, testes' weight, luteinizing hormone (LH) and follicle-stimulating hormone (FSH), testosterone, and lipid profile in passive smokers. The experiment on how *Eruca sativa* leaves affect sperm morphology and concentration is performed by grinding leaves to make the aqueous juice. The research participants were grouped into four groups: a control group, *Eruca sativa-*treated, cigarette-treated, and a group receiving both *Eruca sativa* and cigarette exposure. The rats were weighed and euthanized surgically, and the testes were harvested and weighed after four weeks of treatment. The sperm count was determined using epididymal sperm, and sperm morphology was determined using vas deferens sperm. The collected cardiac blood was used for lipid profile assessment and hormone-level determination. The findings of this study are significant. Tobacco exposure led to a notable increase in abnormal sperm and a decrease in sex hormone levels.

In contrast, the *Eruca sativa* group showed a highly significant difference in sperm morphology and counts compared *to* the cigarette group, with a *p*< 0.001. Although there was a slight decrease in the lipid profile concentration, it was insignificant. Importantly, the co-administration of *Eruca sativa* and cigarette smoke resulted in a significant reduction in abnormal sperm count, increased sperm count, higher sex hormone concentration, and lipid profile. The *Eruca sativa* juice used in this study had a protective effect that could be used to reverse or prevent the effects mentioned above of passive smoking.

## Introduction

Research on medicinal plants by researchers worldwide to understand secondary metabolites and active biomolecules, crucial to knowing the potential of medicinal genera, has attracted their attention [1]. Because the uses of this plant are somewhat similar, traditional medicine remains a primary treatment option in the formal health system in most Arabic countries. One of these therapeutic plants, *Eruca sativa*, also known as 'Rocket' and part of the *Brassicaceae* family, has been used as a traditional therapy for many illnesses [2]. Several plant components of *Eruca sativa* are abundant in fiber and bioactive compounds known to have medicinal and aphrodisiac benefits [3,4]. Due to various plant components, the leaves of this plant are abundant in fiber and bioactive compounds with medicinal and aphrodisiac applications [5]. Phytochemicals reported to be found in this crop include flavonoids, phenolic acids, terpenes, carotenoids, tannins, glycosides, saponins, sterols, alkaloids, and other secondary metabolites [5,6]. The aphrodisiac effect of the *Eruca sativa* plant promotes male fertility and sexual activity through spermatogenic proliferation, seminiferous tubule dilatation, and an increase in sex hormone levels [7]. Moreover, the plant can promote the development of the testes and improve the spermatozoa's proliferation, maturation, and differentiation [8]. Contrarily, the addictive substance known as nicotine is present in cigarettes, which are small rolls of absorbent paper stuffed with a rod of chopped-up tobacco leaves. The kind and section of the leaf that is utilized determine the nicotine level of the cigarette; the higher the leaf is taken, the higher the nicotine content [9]. Smoking continues to be a serious health issue because of nicotine use. It has been linked to decreased fertility, significantly decreased testosterone, luteinizing hormone (LH), and follicle-stimulating hormone (FSH) levels after nicotine administration, and distorted reproductive hormone levels in male rats after nicotine administration. It is conceivable that testicular dysfunction and low testosterone levels are related [10]. Likewise, it is well-documented that cigarette smoke or passive smoking is linked to a decline in pregnancy rates, altering both male and female fertility [11], sperm count and motility [12], and sperm density [13,14]. One of the main causes of dispersal dysgenesis is that it results in Leydig cell malfunction and deficiencies in spermatogenesis and sperm maturation [15,16]. Studies have demonstrated that the degenerative impact of cigarette smoke on testicular tissues, which causes altered sexual behaviors and fertility in male rats, may be prevented by pretreatment with antioxidants like vitamin E, which is also present in *Eruca sativa* leaves [17]. This research aims to explore the impact of nicotine exposure on the fertility of male rats due to the absence of existing studies illustrating how *Eruca sativa* leaves affect rats exposed to cigarette smoke and subsequently treated with a medicinal plant.

## Materials and methods

Preparation of stock solution (*Eruca sativa* solution)

Sixty pieces of fresh *Eruca sativa* leaves were purchased from the local market, originating from Sharazur/Suleimani. They were dried indoors, ground using an electric grinder, and then mixed with distilled water to create a juice stock solution. One tablespoon of *Eruca sativa* powder was mixed with 100 ml of distilled water to prepare the solution, kept for 24 hours at 20°C in the refrigerator, and filtered using filter paper. For each rat, an average of 15.4 ml per body weight of the *Eruca sativa* aqueous solution was administered. The dosage of anesthetic ketamine is 40-100 mg/kg, and xylazine is 5-13 mg/kg. Oral gavage (dosing) was used for the juice administration."

Cigarette method

To expose the rats to smoke, a glass container similar to a fish tank was utilized. Rats aged 8-10 weeks were exposed to a cigarette pack containing 20 cigarettes for one hour, with ten-minute intervals for the rats to inhale fresh air.

Experimental design

We used a total of 16 young adult rats aged 8-10 weeks and weighing about 200g-370g. The Wistar Rats, also known as Rattus norvegicus, were obtained from the College of Veterinary, University of Suleimani. The animal experiments, breeding, and parturitions were conducted in the animal house of the Medical Laboratory of Science, College of Health Sciences, University of Human Development. Each group of treatments was composed of four replicates, with all rats fed with the standard rodent diet, which was comprised of 24.5% barley, 30% wheat meal, 22.5% yellow corn, 15.2% soy, 0.45% salt, 0.2% limestone, and 7.5% animal protein. Ad libitum, drink water at 23±2ºC for 12-12 hours on a light-dark cycle. The plastic cages were washed and changed twice a week.

Sixteen male albino rats (Rattus norvegicus) (n=16) were divided into 4 groups: Control (C) and three treated groups designated as T1, T2, and T3.

Four rats (n=4) in each group were categorized as follows:

C: Rats consuming a regular standard diet only.

T1: Rats treated with 15.4 ml/kg BW of *Eruca sativa* solution (four times/week).

T2: Rats exposed to cigarette smoke daily for four weeks.

T3: Rats were treated with 15.4 ml/kg BW of the *Eruca sativa* solution (four times/week) and also exposed to cigarette smoke daily for four weeks.

Dissection and blood sampling assay

At the end of the treatment period, the rats were rendered unconscious through an intramuscular injection of ketamine/xylazine anesthesia in a 2:1 ratio. The dose of anesthetic ketamine is 40-100 mg/kg, and xylazine is 5-13 mg/kg. Blood samples were then collected via heart puncture and centrifuged at 2500 rpm for 10 minutes to procure the serum sample. After anesthetizing the rats with ketamine/xylazine anesthesia, we dissected the rats.

Sperm morphology assay

The vas deferens were surgically severed from the testes, and semen was carefully extracted onto a clean slide, creating a smear for each sample. These smears were then treated with an eosin stain. Subsequently, the slides were allowed to air-dry, and the outcomes were subsequently examined [18,19].

Epidydemial sperm count

For each rat, a single caudal epididymis was removed, cut into four pieces, and then mixed with 2 ml of normal saline for the semen to leak out. Next, transfer 20 microliters of sperm to an Eppendorf tube containing 20 microliters of normal saline and 10% formalin for 5 minutes. Five microliters of the suspension were then transferred to a clean Neubauer chamber and kept in a wet place for the sperm to settle properly, and the results were read using a light microscope (40X) [20].

Statistical analysis 

The data underwent analysis using the IBM Corp. Released 2019. IBM SPSS Statistics for Windows, Version 26.0. Armonk, NY: IBM Corp. The experimental results were expressed as mean ± standard deviation, and groups were compared by analysis using the ANOVA test. A p≤0.05 was considered indicative of a significant difference.

A post-hoc analysis refers to a statistical analysis specified after a study has been concluded and the data has been collected. Therefore, such tests are also called multiple comparison tests. Post-hoc tests are used as a follow-up to the ANOVA to determine which pairwise comparison of means contributes to the overall significant difference.

Lipid profile and sex hormone tests 

For sex hormone tests, we utilized Roche (Cobas E411) with the principle of electrochemiluminescence. The kits used are Elecsys Testosterone II, Elecsys LH, and Elecsys FSH. The prepared sample is mixed with specific reagents that contain antibodies or antigens that bind to the hormone of interest. The bound antigen-antibody combinations light up with chemiluminescence. When the target hormone is present, specific molecules in the reagents react with one another to produce light. The device uses standards and calibration curves to process the detected light signals and translate them into quantitative output.

In the lipid profile tests, we used Roche (Cobas 6000) with the principle of colorimetry. The kits used are CHOL2, TRIGL, HDLC4, and LDLC3. The sample is treated with particular chemicals in order to make the measurement of several lipid characteristics easier. Enzymes, substrates, and other elements required for the test reactions are included in these reagents. The results of these enzymatic processes, which are commonly quantified as changes in absorbance or fluorescence, are found by the Cobas 6000 analyzer. The target lipid analyte's concentration in the sample determines the signal's strength.

## Results

Lipid profile

In our study, the highest mean levels of cholesterol, high-density lipoprotein HDL, and low-density lipoprotein LDL were recorded in rats treated with *Eruca sativa*, with an estimated mean ± SD = 79.25 ± 12.69, 46.25 ± 6.18, and 17.00 ± 9.42, respectively. The control group exhibited the highest mean level of triglycerides (mean ± SD = 63.75 ± 39.16). Notably, the lowest mean serum levels of all lipid profiles, including cholesterol, triglycerides, HDL, and LDL, were recorded in the group of rats exposed to cigarettes without significant statistical differences (p> 0.05). See Table 1 for a comparison of the lipid profile between the study groups.

**Table 1:** A comparison of the lipid profile between the study groups. NS: No significant deference.

Lipid Profile	Study Groups (16 Rats)					p-Value
	C (n=4)	T1 (n=4)	T2 (n=4)	T3 (n=4)	Total	
Cholesterol mg/dl	64.25 ± 8.73	64.01 ± 12.69	63.50 ± 1.29	66.75 ± 0.96	68.44 ± 9.54	0.061 ^NS^
Triglyceride mg/dl	63.75 ± 39.16	39.75 ± 21.2	37.50 ± 1.29	58.25 ± 3.4	49.81 ± 23.18	0.295 ^NS^
HDL mg/dl	42.25 ± 5.25	46.25 ± 6.18	37.75 ± 4.27	45.25 ± 2.99	42.87 ± 5.5	0.109 ^NS^
LDL mg/dl	14.25 ± 4.03	17.00 ± 9.42	10.50 ± 1.29	13.50 ± 1.29	13.81 ± 5.23	0.404 ^NS^

Sperm morphology

Table 2 presents the sperm morphology of the rats. Regarding normal morphology, we counted about 100 sperm from each slide or animal (400 sperm for each treatment) to determine sperm morphology abnormalities. The highest mean level was recorded in the *Eruca sativa* group (83.75 ± 3.77), while the lowest mean value was in the cigarette group (56.50 ± 7.42), showing a highly significant difference (p < 0.001). Paradoxically, the cigarette group recorded the highest mean level of abnormal morphology (43.50 ± 7.42), while the *Eruca sativa* group recorded the lowest level (16.25 ± 3.77) with a highly significant difference (p < 0.001).

**Table 2:** A comparison of the sperm morphology of the rats between the study groups NS: No significant deference. *: Significant deference. **: Highly significant deference.

Variable	Study Groups (16 Rats)					p-Value
	C (n=4)	T1 (n=4)	T2 (n=4)	T3 (n=4)	Total	
Normal morphology	72.25 ± 5.56	83.75 ±3.77	56.50 ± 7.42	73.25 ± 6.40	71.44 ± 11.37	< 0.001**
Abnormal morphology	31.00 ± 3.83	16.25 ± 3.77	43.50 ± 7.42	26.75 ± 6.40	29.37 ± 11.25	< 0.001 **
No head	3.75 ± 2.87	2.25 ± 0.50	4.50 ± 1.73	3.00 ± 2.71	3.38 ±2.13	0.519 ^NS^
Hook less	2.00 ± 0.82	2.50 ± 0.58	5.25 ± 1.71	2.50 ± 1.29	3.06 ± 1.69	0.008 *
Corkscrew	2.25 ± 0.96	3.00 ± 0.82	5.00 ± 1.41	3.75 ± 2.50	3.50 ± 1.75	0.136 ^NS^
Coiled tail	5.50 ± 2.08	2.75 ± 0.96	6.25 ± 2.75	3.25 ± 3.86	4.44 ± 2.80	0.227 ^NS^
Broken tail	2.75 ± 1.71	2.75 ±0.50	5.00 ± 1.83	1.00 ± 0.82	2.87 ±1.89	0.01 *
Bent tail	8.5 ± 1.29	2.25 ± 2.5	10.75 ± 5.56	7.0 ± 2.94	7.13 ± 4.46	0.027 *
Detached head	3.0 ± 0.82	0.75 ± 0.96	6.75 ± 2.5	6.25 ± 5.06	4.19 ± 3.62	0.039 *

Sperm cell count

As demonstrated in Table 3, the highest mean level of sperm cells was noted in rats treated with both *Eruca sativa* and cigarettes (111.25 ± 6.08). On the other hand, the lowest mean level was in the cigarette group (59.50 ± 1.29), showing a highly significant difference (p < 0.001).

**Table 3:** A comparison of sperm cell count between the study groups.

Variable	Study Groups					p-Value
	C (n=4)	T1 (n=4)	T2 (n=4)	T3 (n=4)	Total	
Sperm cell count (×10⁶ sperm/ml)	65.25 ± 1.71	106.25 ± 7.50	59.50 ± 1.29	111.25 ± 6.08	85.56 ± 24.51	< 0.001 **

Sex hormones

Our study revealed that all study groups have the same value of follicle-stimulating hormone (FSH) (< 0.3 mIU/ml). Regarding luteinizing hormone (LH), the highest mean level was recorded in the control group (0.41 ± 0.02 mIU/ml), and the lowest level was in the cigarette group (0.38 ± 0.1) with a p-value (p = 0.039). Also, the highest serum level of total testosterone was in the control group (3.3 ± 0.41 ng/ml), and the lowest value was noted in the cigarette group (0.38 ± 0.11 ng/ml), with a highly significant difference statistically (p< 0.001). All of these results are listed in Table 4.

**Table 4:** Serum levels of sex hormones in different study groups. *: Significant deference. **: Highly significant deference.

Sex hormones	Study Groups						p-Value
	C (n=4)	T1 (n=4)	T2 (n=4)	T3 (n=4)		Total	
FSH (mIU/ml)	< 0.3 ± 0.00	< 0.3 ±.00		<0.3 ± 0 .00	< 0.3 ± 0.00	<0.3 ± 0.00	/
LH (mIU/ml)	0.30 ± 0.02	0.39 ± 0.1		0.38 ±0.1	0.39 ± 0.1	0.39 ± 0.2	0.039 *
Total Testosterone (ng/ml)	2.23 ± 0.33	2.25 ± 0.32		0.38 ± 0.11	1.62 ±0.11	1.88 ± 1.12	< 0.001 **

Sperm morphology abnormalities

**Figure 1:**
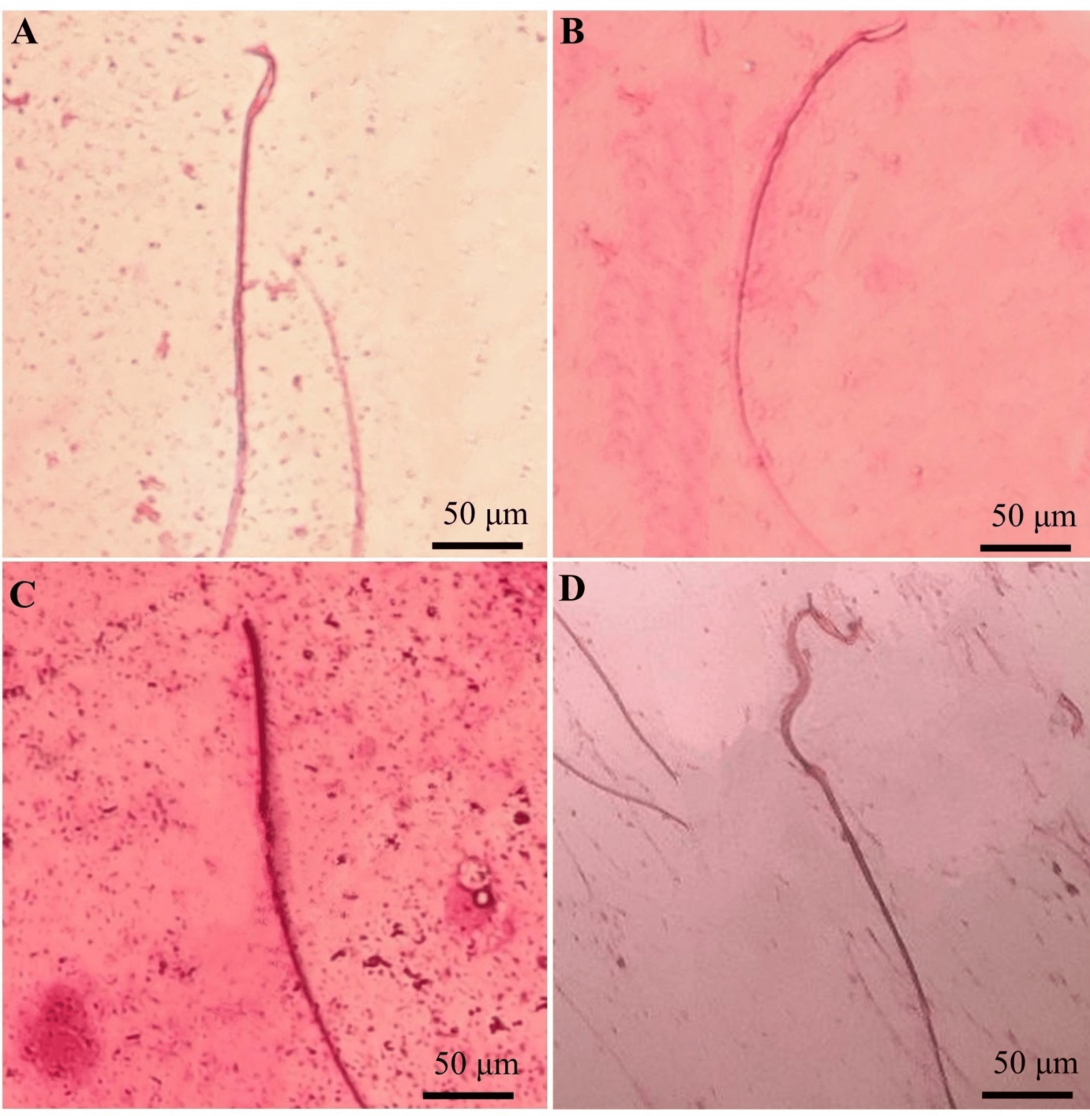
Sperm morphologies: (A) normal, (B) hookless, (C) no head, (D) corkscrew

Many of the toxins in cigarette smoke can be harmful to male reproductive health. Cigarette smoking is indeed a well-known risk factor for reducing average semen production in men [21]. Our study's findings are supported by the aberrant sperm morphologies shown in Figure 1. Rats with nicotine-induced morphological abnormalities include (B) "hookless," which is a sperm without a head, such as an acephalic or decapitated syndrome sperm (C) "no head" sperm that has neither a head nor any appearing structure (D) "corkscrew"; a few other cylindrical objects have twisted midpoints.

**Figure 2:**
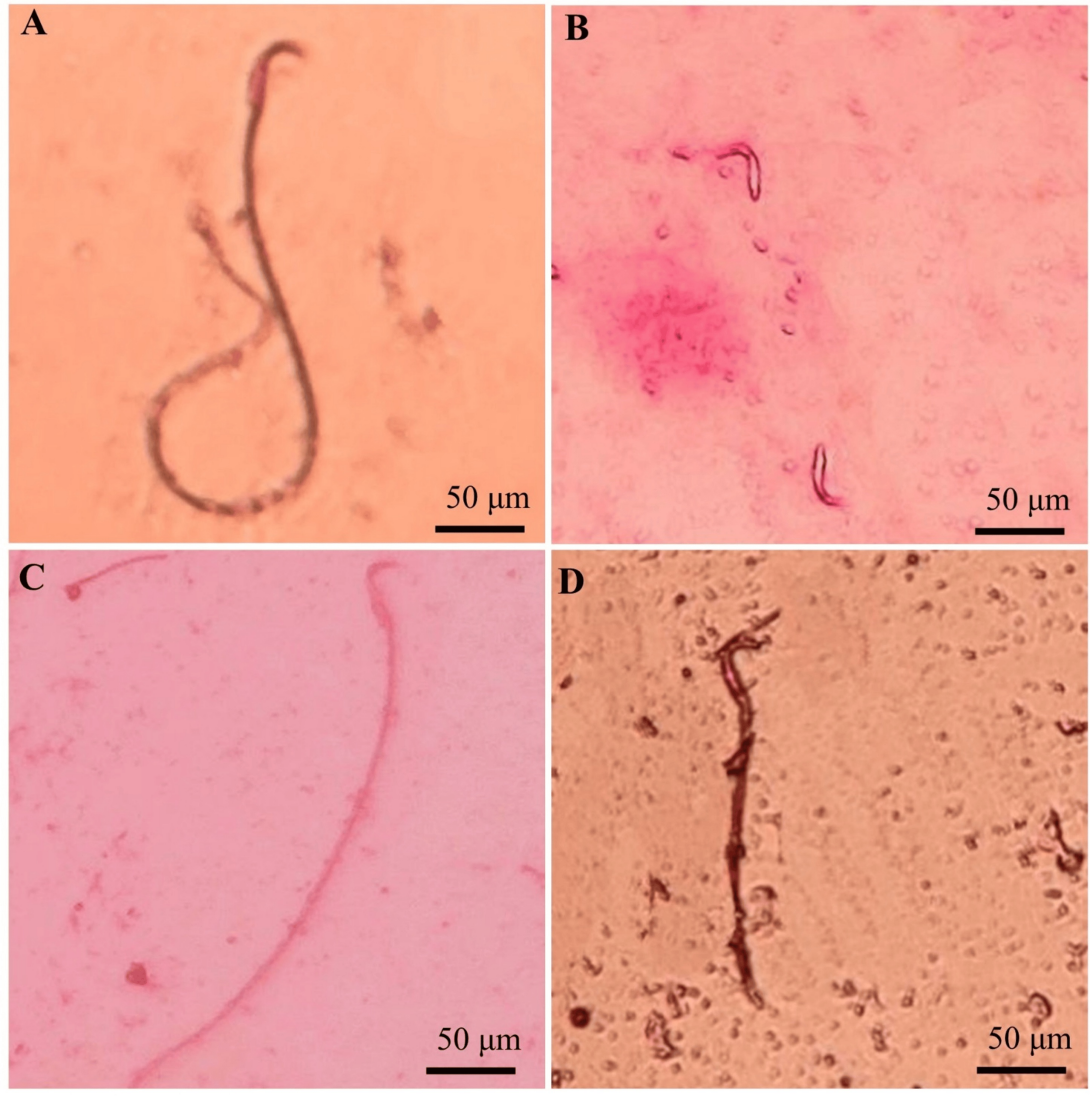
Sperm morphologies (A) coiled tail, (B) detached head, (C) bent tail, (D) broken tail.

Figure 2A-2D shows the different sperm morphologies of (A) "coiled tail," smokers develop mainly from heavy smokers. Sperm resulting from this condition cannot get to their eggs. Simply because their tails are damaged, it does not swim (B) "detached head," and the heads cannot connect to the sperm's tail [22,23] (C) "bent tail" with an internal "droplet." If any sperm has a (D) "broken tail," it functions less far than smokers or haciendas, and sperm does not swim to link to the egg while the tail is missing. A broken tail of sperm refers to the portion of the sperm cell’s structure called the flagellum. If the tail becomes detached, it will impair the sperm’s ability to swim effectively. Motility develops sperm while they are in the epididymis, where these abnormalities are likely to occur [24]. Smoking contributes to oxidative stress since the plasma membrane of smoke-exposed sperm has a large amount of polyunsaturated fatty acids, leading to higher lipid peroxidation [25]. 

## Discussion

In this study, T1 shows a lowered level of triglycerides with a non-significant increase in low-density lipoprotein (LDL) levels. This suggests that the administration of *Eruca sativa* (E.S.) improved the metabolism of adipocytes in mice on a high-fat diet by enhancing citrate synthase activity and lowering triglyceride levels [24]. In T2, rats exposed to cigarette smoke showed increased total cholesterol levels and lowered high-density lipoprotein (HDL) levels, indicating that smoking had an adverse effect on health. A study published in 2003 agreed with the present results, as cigarette smoke exposure increased total cholesterol and decreased high-density lipoprotein-cholesterol (HDL-C) levels. The researchers suggested that oxidative stress and inflammation caused by cigarette smoke may be responsible for these effects on lipid metabolism [25]. While it's widely accepted that smoking generally leads to decreased serum levels of high-density lipoprotein cholesterol (HDL-C) and increased serum triglycerides, some studies have suggested that smoking has a minimal effect on triglyceride levels in males. To further explore this, researchers needed to examine dose-response relationships, particularly as low-density lipoprotein-cholesterol (LDL-C) levels decreased in individuals smoking more than 10 cigarettes daily. The observation that smoking raises cholesterol and lowers high-density lipoprotein-cholesterol (HDL-C) concentrations offers a plausible biological explanation for its effects [21]. This approves the present results demonstrating a non-significant reduction in triglyceride (TG) and LDL levels of the male rats as high doses of cigarettes were administered, which caused an adverse effect on lipid profile parameters. While in T3, both cigarettes and *Eruca sativa* juice were administered parallel to each other, this is considered a first-time trial for such treatment, so no studies have been found to compare the results. Although the result showed an increase in all lipid parameters compared to T1 and T2, this indicates a serious interaction between both plants and cigarette components that caused such an alteration in their effect. This is perhaps due to the dose-response relationship of both cigarettes and *Eruca sativa* juice, which tends to increase HDL, while contrary to all predictions, LDL, triglycerides, and cholesterol levels have risen compared to T1 and T2.

Medicinal plants are natural products recognized for their unique treatment properties and have been employed as remedial agents for many years. However, as medicinal plants contain various bioactive compounds responsible for their activity, using herbal remedies simultaneously with or exclusively with standard medicines can alter their activity. Such an activity is usually called additive, super-additive, or infra-additive impact on the treatment effect. The present research uncovered that sperm abnormal shapes notably improved in T1, demonstrating the lowest abnormal shape compared to the control and all administered groups. The T3-treated groups showed a notable improvement in the incidence of typical sperm morphologies compared to the control group and T2. Cigarette smoke has long been recognized as comprising numerous dangerous substances. Tobacco smoking has been linked to diminished sperm condensation among smokers, including count, strength, motility, viability, and the percentage of typical cells [26,27]. In this work, T2 presented an increase in abnormal sperm morphologies compared to the control groups, T1 and T3. The sensation of spermatozoa to nicotine in this research was seen as significant distortions, such as bent tails, underdeveloped tails, curved midpieces, and bent midpieces. These distortions typically occur when spermatozoa are transiting through the epididymis and being immersed and saved, to a degree that correlates with the purchase of motility or capacity [28]. Smoking in spermatozoa has often caused oxidative stress (OS) by overproducing reactive oxygen species (ROS), mainly due to its high polyunsaturated fatty acid content in its plasma membrane [29].

The tabulated data (Table 3) for groups T1 and T3 provided evidence about the influence of *Eruca sativa* juice on the scavenging system, an essential mechanism in reducing the adverse effects of ROS generated by tobacco cigarette smoke [30]. This explains the role played by *Eruca sativa*'s antioxidant properties through its phenolic compounds, which affect the modification of antioxidant enzymes by improving their function [31]. Furthermore, the seed extract of *Eruca sativa* showed evidence of indirectly stimulating the reproductive gonadal system's function via the androgenic activity pathway. The ability of *Eruca sativa* juice to increase the quantity of Leydig cells could be facilitated by its scavenging function on Fe3+ ions [32]. Again, these findings confirmed previous studies already documented in the literature to attest to the potential of *Eruca sativa*'s capability to enhance sperm quality and fertility [33,34]. The average sperm count of rats in group T1 that were administered with *Eruca sativa* aqueous solution had a statistically significant sperm count quantity compared with those administered with pure water. Hence, the observed growth in testes and spermatozoa proliferation, differentiation, and maturation may be due to the ability of *Eruca sativa* juice to compare to the control group. In the earlier research, it was reported that the majority of the rats showed a statistically significant increment in spermatogenesis, testosterone levels, and sperm function and a statistically significant decrease in the number of sperm that were dead and malformed in Capparaceae leaf juice [7]. All the results corroborate existing research on the abundance of saponin, alkaloids, terpenes, flavonoids, glycosides, and steroids within rocket juice that favor the supply of sperm [35].

Importantly, these compounds have the advantage of requiring low doses for adjuvant activity [36]. Conversely, in Table 3, the rats exposed to cigarette smoke, specifically in the (T2) group, exhibited the lowest mean sperm count when compared to the control group. This decline can be attributed to the direct toxic effects of cigarette smoke on germ cells [37]. Exposure to cigarette smoking significantly affected the rat testes, resulting in smaller organs with a lower absolute testicular weight compared to the control group. It also impaired spermatogenesis, leading to lower daily sperm production, reduced sperm counts, a higher percentage of abnormal sperm, a lower percentage of motile sperm, and reduced testosterone levels [16]. Inhalation of cigarette smoke caused damage to the seminiferous epithelium, with primary spermatocytes and Sertoli cells appearing to be particularly vulnerable. The findings suggest that cigarette smoke inhalation can induce specific disruptions in spermatozoon development, possibly through direct or indirect toxicity to spermatogenesis [37].

Furthermore, the highest average sperm count was observed in rats treated with both *Eruca sativa* juice and exposed to cigarette smoke, specifically in the (T3) group, when compared to the control group as well as the groups treated with *Eruca sativa* aqueous solution (T1) and those exposed to cigarette smoke alone (T2). This suggests that *Eruca sativa* may offer some protective effects against the direct toxicity of cigarette smoke on germ cell structure and function within the testes or in post-testicular organs like the epididymis and vas deferens. In the (T3) group, the elevated sperm count observed may be attributed to the presence of polyphenolic flavonoid compounds [31] and a combination of saponins, alkaloids, terpenes, flavonoids, glycosides, and steroids [35] found in the leaf juice.

The aqueous solution of *Eruca sativa*, as seen in the T1 group, exhibited increased levels of overall sex hormones, except for follicle-stimulating hormone (FSH), which showed no significant impact compared to the control group. These findings suggest that *Eruca sativa* positively influences the hormonal profile, supporting its potential benefits in managing reproductive dysfunction. The higher rise in testosterone and a slight increase in luteinizing hormone (LH) in the T1 group compared to T2 are thought to be associated with the presence of polyphenolic flavonoid compounds in the leaf juice [31]. These antioxidants likely have a protective effect, reducing damage caused by free radicals in the rat testis.

Table 4 displays that rats treated with cigarette smoking (T2) had the lowest mean level of sex hormone profile compared to controls. Compared to the control group, in the T2 group, a significant reduction in testosterone levels was observed in rats exposed to cigarette smoke, as well as a little decrease in LH and FSH hormones. Yamamoto et. al. [16] reported that the rats exposed to cigarette smoke had decreased sperm motility and condensation, impaired Leydig cell function, and reduced hormonal secretions from the genital system. However, they found some discrepancies between their findings and the current outcomes owing to variations in research methods, duration, and dose-response approach used [38]. There are several harmful chemicals found in cigarette smoke, including nicotine and polycyclic aromatic hydrocarbons, which disrupt the endocrine system and interfere with hormone production [39].

Furthermore, these chemicals can damage the cells that produce testosterone and sperm in the testes by causing oxidative stress and inflammation. Smoking has also been linked to reduced testosterone levels, decreased sperm motility, and increased sperm abnormalities in male rats. These effects can lead to infertility and other reproductive problems [40]. *Eruca sativa* seed oil stimulates spermatogenesis and increases testosterone levels in rats, leading to a significant increase in sperm activity and a return to normal levels of testosterone [35]. In a 2016 study assessing the protective effects of *Eruca sativa* seed oil against testicular damage caused by oral nicotine administration in rats [41], the results indicated that *Eruca sativa* played a protective role by nearly completely reversing all morphometric and histological changes induced by nicotine. Similarly, the current findings demonstrate a strong protective effect of *Eruca sativa* against the impact of nicotine, particularly in preventing the decrease in testosterone levels, with a significant increase in testosterone levels reaching normal values. Because human and rat tissues share metabolic similarities, it can be inferred that cigarette smoke might impact the process of sperm development. However, further research on humans is necessary to confirm this. Given the significance of fertility in humans and the widespread prevalence of smoking, particularly among young individuals, smokers should be educated about the adverse effects of cigarette smoking on fertility as a precaution.

The study limitations include the sample size, which is relatively small, and the rat model, which cannot fully transfer to humans. Another significant limitation is the absence of direct human data that should be validated via epidemiological studies and clinical trials. Furthermore, extra studies with larger sample sizes are required to confirm these findings and enhance their applicability to people. 

## Conclusions

In conclusion, *Eruca sativa* aqueous solution significantly protects sperm morphology against tobacco cigarette smoke-induced injury. It also significantly increases the mean level of sperm count. Regarding sex hormone levels, *Eruca sativa* significantly increases testosterone and insignificantly increases luteinizing hormone (LH) levels compared to the cigarette smoke-exposed group, while showing no significant difference in follicle-stimulating hormone (FSH) levels. In terms of lipid profile, *Eruca sativa* couldn't cause any significant difference. These findings suggest the potential protective and beneficial effects of *Eruca sativa* on male reproductive health in the presence of cigarette smoke exposure.
